# Precision laser acupuncture at back-shu points enhances growth performance, feed efficiency, and hormonal regulation in rabbits

**DOI:** 10.14202/vetworld.2025.2367-2376

**Published:** 2025-08-21

**Authors:** Mudhita Zikkrullah Ritonga, Ertika Fitri Lisnanti, Widya Pramita Lokapirnasari, Sri Hidanah, Saifur Rehman, Muhammad Fajar Amrullah, Rico Anggriawan, Mutasem Abuzahra, Denny Irmawati Hasan, Aamir Shehzad, Siti Rani Ayuti

**Affiliations:** 1 Doctoral Program of Veterinary Science, Faculty of Veterinary Medicine, Universitas Airlangga, Surabaya, Indonesia; 2 Laboratory of Anatomy, Faculty of Veterinary Medicine, Universitas Syiah Kuala, Banda Aceh, Indonesia; 3 Center for Tropical Veterinary Studies and One Health Collaboration Center, Universitas Syiah Kuala, Banda Aceh, Indonesia; 4 Program of Animal Husbandry, Faculty of Agriculture, Universitas Islam Kadiri, Kediri, Indonesia; 5 Department of Animal Husbandry, Faculty of Veterinary Medicine, Universitas Airlangga, Surabaya, Indonesia; 6 Department of Microbiology, Faculty of Veterinary and Animal Sciences, Gomal University, Dera Ismail Khan, Pakistan; 7 Doctoral Program of Animal Biomedical Science, School of Veterinary Medicine and Biomedical Sciences, IPB University, Bogor, Indonesia; 8 Program of Animal Husbandry, Faculty of Agriculture, University of Kahuripan Kediri, Kediri, Indonesia; 9 Department of Parasitology, Faculty of Veterinary Medicine, Universitas Airlangga, Surabaya, Indonesia; 10 Laboratory of Pathology, Faculty of Veterinary Medicine, Universitas Syiah Kuala, Banda Aceh, Indonesia; 11 Disease Diagnostic Laboratory Bhakkar, Department of Livestock and Dairy Development, Government of Punjab, Punjab, Pakistan; 12 Laboratory of Biochemistry, Faculty of Veterinary Medicine, Universitas Syiah Kuala, Kota Banda Aceh, Indonesia

**Keywords:** back-shu points, feed efficiency, growth hormone, laser acupuncture, rabbit, veterinary acupuncture

## Abstract

**Background and Aim:**

Laser acupuncture has emerged as a promising non-pharmacological approach to enhance productivity in livestock. This study aimed to evaluate the effects of laser acupuncture applied to specific back-shu points (Bladder [BL]13, BL15, and BL21) on growth performance, feed conversion ratio (FCR), feed efficiency (FE), and growth hormone (GH) levels in rabbits.

**Materials and Methods:**

A total of 24 male Hycole rabbits were randomly assigned to three groups (n = 8): Group A (placebo control), Group B (laser acupuncture on BL13, BL15, and BL21), and Group C (laser acupuncture on non-specific points). Treatments were conducted weekly over a 6-week period using a 0.2-joule semiconductor laser device. Body weight, feed intake, FCR, FE, and GH concentrations were recorded. GH levels were analyzed using an enzyme-linked immunosorbent assay. Data were statistically evaluated using a one-way analysis of variance followed by Duncan’s *post hoc* test.

**Results:**

Group B showed significantly higher final body weight (1.97 ± 0.07 kg), weight gain (919 ± 128 g), GH levels (1.75 ± 0.12 ng/mL), improved FCR (2.67 ± 0.07), and greater FE (37.45% ± 1.09%) compared to Groups A and C (p < 0.05). No significant differences were observed between Groups A and C, indicating the importance of targeting precise acupuncture points for therapeutic effectiveness.

**Conclusion:**

Laser acupuncture at BL13, BL15, and BL21 significantly enhances metabolic efficiency, growth performance, and hormonal regulation in rabbits. The precision of point application is critical for achieving optimal physiological benefits. This technique provides a sustainable and non-invasive approach to enhancing feed utilization and productivity in rabbit farming and holds promise for broader application in modern animal husbandry.

## INTRODUCTION

Acupuncture is a time-honored therapeutic practice applied in both human and veterinary medicine. It operates by stimulating specific anatomical points to influence physiological processes. Clinically, it is utilized to address a range of health conditions, promote animal welfare, and demonstrate promising applications in livestock production. With increasing interest in environmentally friendly and sustainable approaches, acupuncture is gaining recognition as a non-pharmacological method for improving livestock productivity. By modulating nervous, hormonal, and metabolic pathways, acupuncture has the potential to optimize physiological functions in animals. This potential underscores the need for continued scientific research into its role within livestock systems. Physiologically, acupuncture has been shown to enhance intestinal villi development [1], support reproductive function [2], and exert anti-inflammatory effects [3]. Although the underlying mechanisms of acupuncture in rabbits remain partially understood, this study seeks to generate stronger empirical evidence. These observations suggest acupuncture holds broad utility for advancing animal health, performance, and productivity in contemporary livestock operations. To date, few investigations have rigorously assessed the efficacy of laser acupuncture at bladder (BL)13, BL15, and BL21 in enhancing rabbit growth.

Final body weight serves as a critical performance metric in livestock production, as it directly indicates both efficiency and productivity [4]. Growth performance in animals is shaped by genetics, nutritional management, environmental factors, and hormonal activity [5]. Within this framework, acupuncture is emerging as a viable therapeutic modality by modulating the neuroendocrine system. Research suggests that stimulation of targeted acupuncture points can promote the release of growth and digestive hormones vital to metabolism and tissue synthesis [5, 6]. Moreover, acupuncture enhances digestive processes and nutrient assimilation, both of which are fundamental to optimal animal growth [7]. These findings reinforce the physiological influence of acupuncture and its value as a strategic tool to improve productivity in modern livestock systems.

Feed efficiency (FE) is a vital parameter in animal husbandry due to its direct impact on growth performance, production costs, and animal well-being. This efficiency is typically assessed through feed conversion ratio (FCR) and FE metrics. FCR reflects the quantity of feed required to achieve 1 kg of body weight gain, whereas FE quantifies how effectively feed is transformed into body mass [8]. Among these, FCR is widely recognized as the principal index for evaluating feed utilization efficiency [9]. Enhancing FE not only lowers production costs but also supports better animal health and welfare [10]. Improvements in this area can significantly boost profitability for livestock producers [11]. Accordingly, exploring various strategies to enhance FE – including acupuncture – becomes essential. Liu *et al*. [12] have demonstrated that acupuncture can elevate FE by increasing digestive enzyme secretion and optimizing gut microbial balance. Therefore, acupuncture may offer an effective, low-impact means to minimize feed wastage and improve production outcomes.

Beyond its influence on growth and FE, acupunc-ture also contributes to the regulation of growth hormone (GH) levels [6]. This hormone regulates the metabolism of proteins, fats, and carbohydrates, and plays a crucial role in the growth of livestock [13]. Ensuring proper hormonal balance is fundamental to achieving optimal development in farm animals. Previous research by Cui *et al*. [14] has shown that acupuncture stimula- tion may elevate GH secretion, thereby potentially promoting growth. However, as the existing literature presents varying outcomes, the present study aims to provide more definitive evidence using a validated enzyme- linked immunosorbent assay (ELISA) method.

Despite the longstanding use of acupuncture in veterinary medicine, its application in livestock production remains underexplored, particularly in the context of precision-based approaches such as laser acupuncture. Previous studies by Ritonga *et al*. [1], Engler and Rodrigues [2], and Nurwati *et al*. [3] have shown that acupuncture can positively influence physiological parameters, including immune function, reproductive health, and nutrient utilization. However, most of this evidence comes from studies involving traditional needling methods, with limited focus on laser acupuncture – a safer, non-invasive, and more consistent alternative. While some reports suggest acupuncture may enhance growth and metabolic regulation in animals, there is a notable lack of empirical data specifically addressing the effects of laser acupuncture on growth performance, FE, and hormonal profiles in livestock. Furthermore, among the few existing studies, inconsistencies in acupuncture point selection and methodological variations limit the comparability and reproducibility of results. In particular, the physiological efficacy of stimulating the back-shu points BL13, BL15, and BL21 – points associated with respiratory, cardiac, and digestive functions in Traditional Chinese Veterinary Medicine (TCVM) – has not been rigorously evaluated in rabbit models. Consequently, the scientific basis for incorporating laser acupuncture into livestock management practices remains insufficiently established. Addressing this knowledge gap is essential to validate acupuncture as a complementary strategy for enhancing sustainable animal production systems.

**Figure 1:**
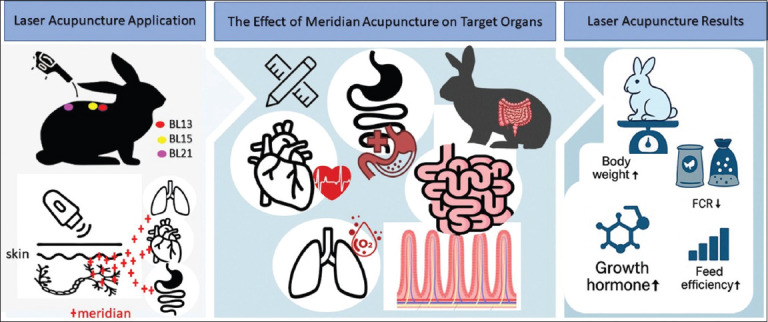
Graphical abstract.

This study aims to comprehensively evaluate the physiological effects of precision laser acupuncture at the BL13, BL15, and BL21 back-shu points in Hycole rabbits, with a focus on growth performance, FCR, FE, and circulating GH levels. By comparing outcomes between animals treated at anatomically correct acupuncture points, non-specific points, and a placebo control, the study seeks to determine whether targeted laser stimulation can yield measurable improvements in metabolic efficiency and production performance. This research intends to offer robust scientific evidence supporting the integration of laser acupuncture into modern livestock management, particularly as a sustainable, non-pharmacological intervention for improving productivity. Furthermore, it contributes to the growing body of literature on the biological mechanisms underlying acupuncture’s effects in livestock, potentially paving the way for broader applications across various species and production settings. The graphical abstract is presented in Figure 1.

## MATERIALS AND METHODS

### Ethical approval

This study was approved by the Animal Ethics Commission of the Faculty of Veterinary Medicine, Universitas Airlangga, Indonesia (Certificate No. 2.KEH.128.09.2022). All experimental procedures were conducted in accordance with Institutional Animal Welfare Guidelines at the Animal Laboratory of the Faculty of Veterinary Medicine, Universitas Airlangga.

### Study period and location

The study was conducted from September to December 2022 at Animal Experimental Unit, Faculty of Veterinary Medicine, Airlangga University, Mulyorejo District, Surabaya City, East Jawa.

### Experimental animals and study design

A total of 24 clinically healthy male Hycole rabbits, aged 4 weeks and weighing 1046 ± 79 g, were used in the experiment [15]. The rabbits were acclimatized for 1 week before treatment initiation. During this period, daily health checks were performed. After acclimation, the animals were randomly divided into three treatment groups (n = 8 per group) using a completely randomized design:

- Group A (Placebo control): Received inactive laser acupuncture at the designated acupuncture points
- Group B (Correct points): Received 0.2-joule laser acupuncture applied precisely at the designated acupuncture points
- Group C (Incorrect points): Received 0.2-joule laser acupuncture applied near – but not directly on – the intended acupuncture points [16, 17].

### Housing and feeding management

Each rabbit was individually housed in an iron wire cage (45.5 × 37.5 × 30 cm) under natural tropical lighting conditions (12-h light/12-h dark cycle), with an ambient temperature of 27°C ± 3°C and relative humidity of 55%–65%. Cages and equipment were cleaned daily and disinfected twice during the study period.

Rabbits were fed a commercial pellet diet formulated according to Indonesian National Standards [18], administered twice daily at 08:00 and 15:00. Clean drinking water was supplied *ad libitum* through nipple bottles. The nutritional composition of the feed is presented in Table 1. Feed intake was recorded daily by weighing both the feed offered and the residuals, with uneaten feed discarded after measurement. Weekly summaries of feed consumption were compiled for the entire 6-week trial.

**Table 1:** Commercial feed analysis.

Component	Quantity
Dry matter	91.16%
Ash	10.3%
Digested energy	2600 kcal
Crude protein	19.09%
Crude fiber	21.58%
Crude fat	3.03%
Total digestible nutrient content	72%
Calcium	12.0 g/kg
Phosphor	6.0 g/kg

### Laser acupuncture protocol

Laser acupuncture treatment was performed once per week for 6 weeks (a total of six sessions per rabbit). A VLP-100 semiconductor laser acupuncture device (20 mW; Biophysics Laboratory, Universitas Airlangga) was used for the treatment. The device was preset to deliver an energy dose of 0.2 joules and automatically shut off when this dose was reached. During each session, rabbits were comfortably restrained in a calm environment to avoid stress.

### Acupuncture point identification

**Figure 2:**
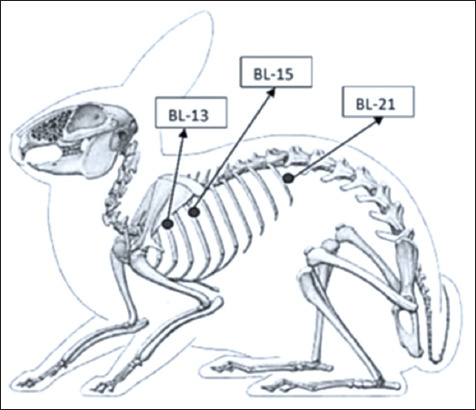
Location of Bladder 13 (BL13), Bladder 15 (BL15), and Bladder 21 (BL21) points [19–22].

Acupuncture stimulation was targeted at back-shu meridian points (Figure 2) [19], specifically:

- BL13 (Feishu): Located 1.5 inches lateral to the third thoracic vertebra; associated with the lung meridian [20]
- BL15 (Xinshu): Located 1.5 inches lateral to the fifth thoracic vertebra; associated with the heart meridian [21]
- BL21 (Weishu): Located 1.5 inches lateral to the thirteenth thoracic vertebra; linked to the digestive tract meridian [22].

Point localization was verified using an acupuncture point detector to ensure accuracy before laser application [19].

### Hormonal analysis and FE evaluation

At 75 days of age, blood samples (3 mL) were collected in the morning from the auricular vein of each rabbit using ethylenediaminetetraacetic acid-coated tubes (Vaculab EDTA K3, OneMed, Surabaya, Indonesia). GH concentrations were measured using an ELISA kit (Elabscience GH ELISA kit, Elabscience Biotechnology Inc., Houston, TX, USA), following the manufacturer’s protocol [23]. The kit had a sensitivity of 0.94 ng/mL and a detection range of 1.56–100 ng/mL, with a high linearity (R^2^ = 0.999). The optical density values of the zero standard and blank were 0.200 and 0.012, respectively, confirming assay sensitivity and reliability.

FCR was calculated as:

FCR = Total feed consumed (g)/Final body weight (g)

FE was calculated as:

FE (%) = (Final body weight/Total feed consumed) × 100 [24].

### Statistical analysis

Data are expressed as mean ± standard deviation. Normality and homogeneity of variance were verified before statistical testing. One-way analysis of variance (ANOVA) was performed to assess differences among groups, followed by Duncan’s multiple range test for *post hoc* comparisons. All statistical analyses were performed using Statistical Analysis System software (SAS Institute Inc., SAS Campus Drive, North Carolina, USA), with a significance level set at p < 0.05.

## RESULTS

### Growth performance

This study assessed the effects of laser acupuncture therapy on rabbit growth by analyzing final body weight across three experimental groups. The rabbits in Group B (acupuncture at precise BL13, BL15, and BL21 points) exhibited the highest final body weight (1.97 ± 0.07 kg), significantly greater than both Group A (placebo control; 1.81 ± 0.12 kg) and Group C (incorrect points; 1.83 ± 0.08 kg) (Table 2). One-way ANOVA revealed highly significant differences in body weight among the groups (p < 0.01), and Duncan’s *post hoc* analysis confirmed that Group B differed significantly from Groups A and C, while no significant difference was observed between Groups A and C.

**Table 2:** A similar pattern was observed for growth hormone levels based on the *post hoc* analysis.

Treatments	Initial body weight (kg)	Final body weight (kg)	Weight gain (gr)	Growth hormone (ng/mL)
A	1.036 ± 0.087	1.810^a^ ± 0.12	774^a^ ± 104.29	1.42^a^ ± 0.08
B	1.051 ± 0.099	1.970^b^ ± 0.07	919^b^ ± 128.02	1.75^b^ ± 0.12
C	1,052 ± 0.059	1.830^a^ ± 0.08	778^b^ ± 100.37	1.51^a^ ± 0.11

^a,b^Different superscripts in the same column indicate significant values (p < 0.05)

### GH levels

GH levels mirrored the pattern observed in body weight. Group B showed the highest GH concentration (1.75 ± 0.12 ng/mL), which was significantly greater than that of both Group A (1.42 ± 0.08 ng/mL) and Group C (1.51 ± 0.11 ng/mL) (Table 2). The *post hoc* test indicated a statistically significant difference between Group B and the other groups (p < 0.01), while Groups A and C did not differ significantly. These results suggest that targeted laser acupuncture at back-shu points effectively enhances GH secretion in rabbits.

### FCR

The FCR values also varied significantly between groups. Group B recorded the lowest FCR (2.67 ± 0.07), indicating the most efficient feed-to-body mass conversion (Table 3). In contrast, Group A had the highest FCR (3.05 ± 0.13), reflecting inefficient feed utilization under placebo conditions. Group C exhibited an intermediate FCR (2.93 ± 0.13), which was not significantly different from that of the placebo group, suggesting that incorrect targeting of acupuncture points may compromise the benefits of laser acupuncture.

**Table 3:** The effects of laser acupuncture treatment on feed intake, FCR, and feed efficiency.

Treatments	Feed intake (g)	FCR	Feed efficiency (%)
A	5511.625^a^ ± 160.67	3.05^a^ ± 0.13	32.82^a^ ± 1.53
B	5284.1245^b^ ± 187.80	2.67^b^ ± 0.07	37.45^b^ ± 1.09
C	5381.25^b^ ± 220.84	2.93^a^ ± 0.13	34.13^a^ ± 1.56

^a,b^Different superscripts in the same column indicate significant values (p < 0.05). FCR=Feed conversion ratio

### FE

FE, calculated as a percentage, further supported the findings from FCR. The highest FE was observed in Group B (37.45% ± 1.09%), followed by Group C (34.13% ± 1.56%), and the lowest in Group A (32.82% ± 1.53%) (Table 3). *Post hoc* comparisons confirmed a significant difference between Group B and the other two groups, while no significant difference was found between Groups A and C.

Collectively, the results demonstrate that precise laser acupuncture at BL13, BL15, and BL21 significantly improves growth performance, FE, and hormonal regulation in rabbits. The absence of significant differences between the placebo and incorrect-point groups highlights the importance of anatomical accuracy in acupuncture application. These findings underscore the therapeutic potential of laser acupuncture as a non-invasive strategy for enhancing livestock productivity.

## DISCUSSION

### Therapeutic relevance of laser acupuncture in livestock

Laser acupuncture is increasingly recognized as a safe, non-invasive, and practical method for stimulating specific points that influence biological functions in both humans and animals [19, 22, 25]. Its simplicity of application and minimal discomfort make it particularly appealing for veterinary and livestock use. This technique targets specific points along the BL meridian – BL13, BL15, and BL21 – known as the back-shu points, which are believed to regulate energy (Qi) flow and the functions of associated organs [19, 22]. Accurate stimulation of these points is essential for promoting energy balance and enhancing metabolic and digestive processes, thereby supporting animal growth and health [7, 10, 26].

### Effect on final body weight

The average body weight in Group B (correct acupuncture) was significantly higher (p < 0.05) than in Groups A and C. These findings indicate that laser acupuncture stimulation meaningfully improves growth performance in rabbits. Ashour *et al*. [27] reported that New Zealand white rabbits fed probiotic strains reached 1562.22 g in winter and 1361.11 g in summer. In addition, Brahmantiyo *et al*. [28] found that Hycole rabbits achieved an average slaughter weight of 2,111.9 ± 462.3 g at 14 weeks of age.

Compared to the control (Group A), the final body weight in Group B was approximately 8% higher and significantly different from both Groups A and C. These results suggest that laser acupuncture may be more effective than dietary probiotics for enhancing rabbit body weight. Probiotics and enzymes are often incorporated into rabbit diets due to their pseudo-ruminant digestion, which relies on hindgut microbial fermentation [29, 30].

This study supports that laser acupuncture at BL13, BL15, and BL21 substantially enhances body weight gain in rabbits, indicating its potential to improve livestock productivity. Increased digestibility promotes better feed utilization within the digestive tract [31]. Moreover, laser acupuncture enhances energy flow to digestive organs [26] and boosts blood circulation [32, 33], which, in turn, improves metabolic efficiency. Greater blood flow allows better oxygen and nutrient delivery to the gastrointestinal tract, facilitating regeneration of intestinal epithelium and gastric mucosa [34].

Consequently, rabbits treated with correctly targeted laser acupuncture gained more weight than those in untreated or incorrectly treated groups. Suboptimal gains in the latter groups underscore the importance of accurate point selection and consistent stimulation [35]. These findings affirm the effectiveness of laser acupuncture in promoting rabbit growth and health. The enhanced weight gain is attributable to improved digestion and nutrient absorption [36], providing strong evidence that laser acupuncture improves both FE and growth outcomes.

### Effect on FCR

Group B demonstrated the lowest FCR, confirming superior FE compared to Groups A and C. Czech *et al*. [37] reported an average FCR of 3.62 in rabbits consuming fermented feed containing *Bacillus subtilis* strain 87Y. Similarly, rabbits supplemented with silver nanoparticles at a 0.2 dose exhibited an FCR of 2.69 [29]. In this study, Group A showed the highest FCR, while Group C had an intermediate value.

FCR is a standard metric for evaluating how effectively livestock convert feed into body mass [38]. Lower FCR values represent more efficient feed use. The lower FCR in Group B suggests that these rabbits converted feed into body weight more effectively than those in Groups A and C. This reinforces the idea that laser acupuncture at BL13, BL15, and BL21 can enhance FE by maintaining stable intake while reducing waste.

Quaresma *et al*. [39] reported that low FCR is associated with high-quality feed, favorable digestive conditions, and effective management. According to Lai *et al*. [40], acupuncture can modulate digestive hormone secretion and stimulate intestinal mucosal growth, thereby enhancing nutrient absorption [41]. These physiological responses reduce the quantity of feed required to support optimal growth.

Group B’s lower FCR occurred without any increase in feed consumption, indicating that acupuncture improved digestive function and nutrient absorption efficiency. Across all groups, feed intake was relatively similar, implying that the metabolic benefits stemmed from acupuncture rather than increased consumption. Rabbits in Group B, treated at the correct acupuncture points, achieved better FCR outcomes, supporting the claim that point accuracy is crucial for improved metabolic performance.

According to Wang *et al*. [6], improved FE following acupuncture is linked to reduced intestinal motility, which increases contact time for nutrient absorption and stimulates epithelial hypertrophy and hyperplasia. Enlarged villi [1] extend the surface area for absorption, enhancing digestion. Reduced motility also slows transit, allowing for prolonged enzymatic action. Digestive hormone and enzyme elevation further enhance metabolic efficiency, enabling optimal growth without excess feed intake.

High FCR values suggest increased feed consumption with lower conversion efficiency, a sign of poor digestibility. Feed consumption is a critical factor in livestock profitability. Al-Sagheer *et al*. [42] emphasized that adequate feed intake ensures optimal energy and nutrient acquisition for growth. The observed improvement in FE in Group B rabbits is likely due to increased digestibility and energy flow resulting from laser acupuncture stimulation [26].

Cho *et al*. [34] explained that stimulation of back-shu points, including BL13, BL15, and BL21, enhances gastrointestinal performance and nutrient absorption. When digestion is efficient, feed waste is minimized, and animals grow optimally. Feed utilization improves, resulting in reduced consumption and stabilized FCR values [43]. Environmental stability and consistent feed quality further support these effects [10].

Findeisen *et al*. [44] also show that acupuncture enhances food metabolism without increasing feed intake. Thus, laser acupuncture can potentially reduce feed demand while maintaining growth and physiological function.

### Effect on FE

FE in Group B was significantly higher (p < 0.05) than in Groups A and C. No significant difference was observed between Groups A and C. This improvement in Group B suggests that laser acupuncture enhances the efficiency of nutrient use. Accurate point targeting enhances nutrient absorption and digestive efficiency, resulting in elevated FE values [10].

Acupuncture may influence the enteric nervous system by promoting mucosal regeneration and intestinal growth through neural stimulation [45]. These effects validate the use of BL13, BL15, and BL21 to improve feed utilization in rabbits. Laser acupuncture-treated rabbits exhibited greater weight gains, suggesting improved gut function and digestibility [36].

Higher FE is also associated with reduced intestinal motility, extending retention time in the lumen for more complete digestion [6]. This mechanism ensures optimal nutrient uptake even with lower feed volumes. Efficient digestion can also reduce the occurrence of gastrointestinal disturbances [45].

According to Ni *et al*. [46], acupuncture modulates intestinal activity, improving digestibility. FE is especially influenced by crude fiber fermentation in the hindgut, which requires optimal microbial activity [47]. Laser acupuncture may enhance microbial activity and stimulate cellulase production, enabling rabbits to extract more energy from fibrous material [48].

In contrast, the lower FE observed in Groups A and C may result from absent or misdirected stimulation, impairing nutrient absorption [5]. These findings support the hypothesis that precise laser acupuncture enhances digestive capacity and feed utilization in rabbits.

### Effect on GH regulation

GH levels followed trends similar to body weight, FCR, and FE. Group B showed significantly higher GH levels (p < 0.05) compared to Groups A and C. No statistical difference was noted between Groups A and C. These results suggest that laser acupuncture at BL13, BL15, and BL21 effectively stimulates GH secretion.

GH, secreted by the pituitary gland, is vital for regulating metabolism, tissue development, and immune responses. Acupuncture may promote GH secretion by modulating the neuroendocrine-immune axis. This complex involves hypothalamic activation, neurotransmitter modulation, and cytokine regulation to enhance hormonal output [14].

Laser acupuncture activates meridian pathways [49], increasing endocrine function and supporting homeostasis in physiological systems [26]. It also promotes metabolic activity by regulating both nervous and hormonal systems [50].

### Mechanistic role of back-shu point stimulation

In TCVM, BL13, BL15, and BL21 are key back-shu points believed to correspond to the lungs, heart, and digestive system, respectively [19]. Stimulating these points is thought to improve Qi flow, influencing internal organ performance [7, 26].

Each point has distinct physiological impacts:

- BL13 (Feishu): Corresponds to the lung meridian. Its stimulation can improve pulmonary function through sympathetic activation, enhancing oxygena-tion and CO_2_ exchange [10, 19, 51]. According to TCVM, lung Qi also affects spleen and stomach func- tion, indirectly benefiting digestion [19]
- BL15 (Xinshu): Associated with the heart meridian. Stimulation may enhance cardiac function, strengthen myocardial contractions, and increase cardiac output [6, 21]. Improved circulation ensures better oxygen and nutrient delivery to the digestive tract [19, 52]
- BL21 (Weishu): Related to the digestive meridian. Stimulating this point can improve gastric motility, enzyme mixing, and nutrient absorption [6, 53, 54]. Enhanced digestion contributes directly to improved growth and FE [26].

Precise stimulation of these back-shu points regulates sympathetic neural pathways that influence visceral organ function, blood flow, and nutrient assimilation [6, 34, 55]. Moon *et al*. [55] emphasized that anatomical and physiological differences among back-shu points necessitate precise targeting for therapeutic success.

## CONCLUSION

This study provides compelling evidence that precision laser acupuncture at BL13, BL15, and BL21 significantly enhances growth performance, FE, and GH regulation in rabbits. Rabbits treated at anatomically correct points (Group B) demonstrated a notable increase in final body weight (1.97 ± 0.07 kg), reduced FCR (2.67 ± 0.07), improved FE (37.45% ± 1.09%), and elevated GH levels (1.75 ± 0.12 ng/mL) compared to the placebo (Group A) and inaccurately treated (Group C) groups. These findings underscore the physiological relevance of accurate acupuncture point localization and support the potential of laser acupuncture as a targeted, non-invasive, and drug-free strategy to optimize livestock productivity.

From a practical perspective, this technique can be incorporated into sustainable rabbit farming systems to reduce feed costs, improve growth performance, and minimize the need for pharmacological growth promoters. The ease of application and animal-friendly nature of laser acupuncture further enhance its utility in commercial animal husbandry.

The strengths of this study include a well-defined experimental design, the use of standardized laser dosages, clearly defined back-shu acupoints, and the integration of both physiological (GH levels) and production metrics (FCR, FE, and body weight) to validate outcomes. The study also adds novel insights by confirming the biological specificity of acupoint stimulation, a concept often neglected in veterinary acupuncture research.

However, the study is limited by its sample size (n = 24), short duration (6 weeks), and focus on a single species (rabbits). These constraints limit the generalizability of the findings to other species and long-term production contexts.

Future research should involve larger animal populations and evaluate this intervention across multiple livestock species (e.g., poultry, goats, and cattle) under varied environmental conditions. Investigations into the molecular and endocrine mechanisms of acupuncture’s action, including transcriptomic and metabolomic profiling, would further strengthen the scientific foundation of this technique. In addition, combining laser acupuncture with other precision farming technologies (e.g., automated monitoring systems and probiotics) may yield synergistic benefits worth exploring.

In summary, laser acupuncture at back-shu points represents a promising and scientifically supported innovation for enhancing livestock performance. With further validation, it can be positioned as a sustainable adjunct to conventional feeding and health management practices, contributing to more efficient and welfare-conscious livestock production systems.

## AUTHORS’ CONTRIBUTIONS

MZR: Supervised field sampling, data collection, laboratory work, and data entry. MZR, WPL, and SH: Conceived, designed, and coordinated the study. EFL, RA, and SRA: Field sampling and data collection. SR, MFA, MA, AS, and DIH: Statistical analysis and interpretation. All authors have read and approved the final manuscript.
